# Complete genome sequence of *Achromobacter anxifer* S12, an aromatic compound-reducing microbe isolated from livestock wastewater

**DOI:** 10.1128/mra.00047-26

**Published:** 2026-06-11

**Authors:** Ye-Ji Hwang, Young Ho Nam, Young Taek Oh, Kook-Il Han

**Affiliations:** 1 Nakdonggang National Institute of Biological Resources (NNIBR) 443035, Sangju, Gyeongsangbuk, Republic of Korea; University of Maryland School of Medicine, Baltimore, Maryland, USA

**Keywords:** livestock odor, odor reduction, livestock environment improving agents

## Abstract

*Achromobacter anxifer* S12 was isolated from livestock wastewater. Here, we report the complete genome sequence of strain S12. Its genome consists of a circular 6.84 Mbp chromosome and a 275 kb plasmid, with a G + C content of 66.0% and encompasses 6,644 protein-coding sequences.

## ANNOUNCEMENT

*Achromobacter anxifer* is a gram-negative, aerobic bacterium belonging to the family Alcaligenaceae and has been isolated from diverse environmental sources, including soil, water, and wastewater (1, 2). Members of the genus *Achromobacter* are recognized for their roles in opportunistic pathogenicity and the bioremediation of diverse environmental contaminants, including aromatic and volatile organic compounds (3–5).

Here, we report the genome sequence of *Ac. anxifer* S12, isolated from livestock wastewater collected at a pig farm in Sangju City, Gyeongsangbuk-do, Republic of Korea (36°25′N, 128°18′E) in October 2022. For enrichment, 5 g of slurry was inoculated into minimal salt medium (MSM; per liter: 3.5 g K₂HPO₄, 1.5 g KH₂PO₄, 0.27 g MgSO₄, 1 g NH₄Cl, 0.03 g Fe₂(SO₄)₃·7H₂O, and 0.03 g CaCl₂) and incubated at 30°C with shaking at 150 rpm for 2 weeks under aerobic conditions. The enrichment culture was serially diluted (10^−1^–10^−10^) and spread on tryptic soy agar (TSA) and then incubated at 30°C for 48 h. A pure isolate was obtained through repeated subculturing.

For genomic analysis, strain S12 was cultivated on TSA at 30°C for 3 days. Genomic DNA was extracted using the Maxwell RSC Tissue DNA Kit (Promega, USA). Long-read sequencing libraries were prepared using the SMRTbell Express Kit (PacBio) following DNA shearing with a g-TUBE (Covaris, USA). Size selection was performed to remove fragments below 15 kb using the BluePippin system (Sage Science, USA). Sequencing was conducted on the PacBio Sequel System, and raw subreads were quality-controlled and adapter-trimmed using SMRT Link v10.0 (6). Short-read sequencing was performed using the TruSeq Nano DNA Kit on the Illumina platform (Illumina, USA), and raw reads were quality-controlled using Trimmomatic v0.39 (7). Genome assembly was performed using PacBio long reads with Flye v2.9 (8). Illumina short reads (HiSeq 2000) were used for two rounds of polishing with Pilon v1.24 (9) and for quality assessment by mapping-based evaluation of coverage and residual errors using BWA-MEM v0.7.17 (10). Default parameters were used except where otherwise noted.

PacBio sequencing generated 93,177 reads (N50, 10,940 bp), totaling 808.9 Mb, whereas Illumina sequencing produced 7,440,467 paired-end reads (2 × 150 bp), with a total yield of 2.2 Gb. *de novo* assembly of the long reads was conducted using Canu v1.7 (11). Assembly quality was assessed by read-depth statistics and evidence of replicon circularization, yielding an overall coverage of 195×. Genome annotation was conducted with the NCBI Prokaryotic Genome Annotation Pipeline (PGAP) v6.10 using GeneMarkS-2+ (12). Default parameters were used except where otherwise noted. Taxonomic assignment was further confirmed by average nucleotide identity (ANI) analysis. The genome sequence of strain S12 exhibited 98.96% ANI, with 87.9% coverage against the type genome of *Ac. anxifer* NCTC 13904ᵀ (GenBank accession no. GCA_900635785.1), significantly exceeding the species boundary threshold of 95%–96%.

The final assembly comprised two circular replicons: a chromosome of 6,838,473 bp and a plasmid (pS12) of 274,700 bp, with a total genome size of 7,113,173 bp (Table 1). The G + C content was 66.0% for the chromosome and 58.5% for the plasmid. The genome contains 6,755 genes, including 6,644 protein-coding genes.

**TABLE 1:** Summary of the assembly and annotation statistics for *Ac. anxifer* S12

Parameter data	
Genetic element	Chromosome + plasmid
Chromosome length (bp)	6,838,473
Plasmid pS12 length (bp)	274,700
Total genome size (bp)	7,113,173
GC content (%)	66.0 (chromosome), 58.5 (plasmid)
No. of coding sequences	6,644
No. of genes (total)	6,755
Sequencing depth (×)	195
GenBank accession no. (assembly)	GCA_053878595.1
RefSeq accession no. (assembly)	GCF_053878595.1
GenBank accession no.	JBSRNO000000000

**Fig 1:**
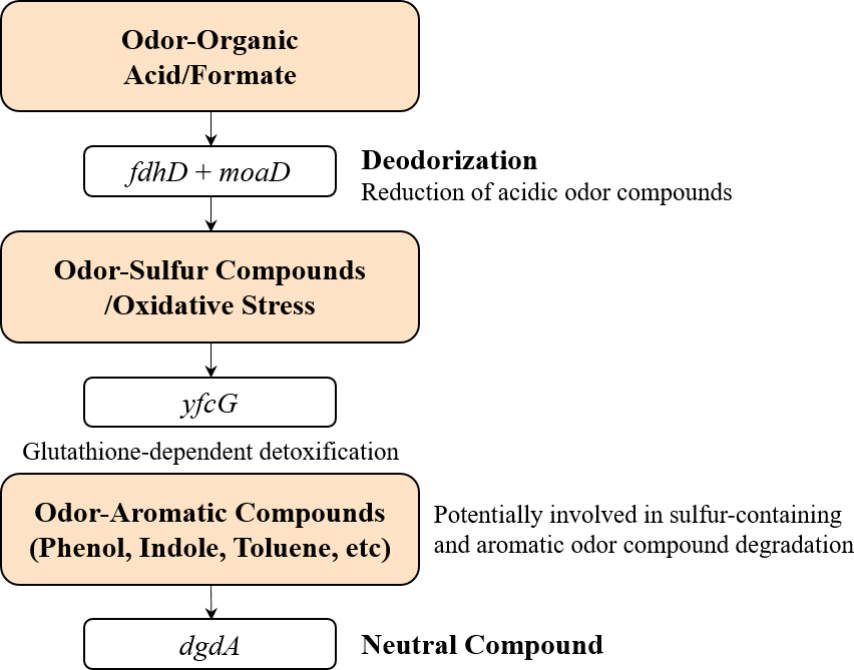
Putative gene-based pathways associated with degradation of odor-related organic acids, sulfur-containing compounds, and aromatic compounds in *Ac. anxifer* S12. Genes identified in the genome are shown as potentially involved in deodorization and detoxification processes.

Strain S12 possesses genes for degrading organic acids (*fdhD* and *moaD*), sulfur compounds (*yfcG*_1), and aromatic rings (*dgdA*) (Fig. 1). These genomic features underpin its potential for odor compound bioremediation. CheckM v1.2.4 analysis indicated 96.47% completeness and 2.04% contamination (13).

## Data Availability

The complete genome sequence of *Achromobacter anxifer* strain S12 has been deposited at the NCBI under BioProject accession PRJNA1303740. The genome assembly is available under GenBank accession GCA_053878595. The whole-genome shotgun project has been deposited under accession JBSRNO000000000 (JBSRNO010000001–JBSRNO010000002). Individual chromosome and plasmid sequences are available under GenBank accessions JBSRNO010000001 (chromosome) and JBSRNO010000002 (plasmid pS12). Raw sequencing data have been deposited in the Sequence Read Archive (SRA) under BioSample accession SAMN50535600, SRA study accession SRP614689, SRA sample accession SRS26339087, and SRA experiment accessions SRX30290418 (PacBio Sequel II) and SRX30290419 (Illumina HiSeq 2000), with corresponding SRA run accessions SRR35185714 and SRR35185713, respectively. All data are publicly available.
